# Microbiological characteristics of smoked and smoked–dried fish processed in Benin

**DOI:** 10.1002/fsn3.1030

**Published:** 2019-04-22

**Authors:** Dona Gildas Hippolyte Anihouvi, Yénoukounmè Euloge Kpoclou, Marleen Abdel Massih, Ogouyôm Herbert Iko Afé, Mahunan François Assogba, Melvina Covo, Marie‐Louise Scippo, Djidjoho Joseph Hounhouigan, Victor Anihouvi, Jacques Mahillon

**Affiliations:** ^1^ Laboratory of Food and Environmental Microbiology, Faculty of Bioscience Engineering UCLouvain Louvain‐la‐Neuve Belgium; ^2^ Laboratory of Food Sciences, Faculty of Agronomic Sciences University of Abomey‐Calavi Abomey-Calavi Benin; ^3^ Laboratory of Food Analysis, Faculty of Veterinary Medicine ULiège, Sart‐Tilman Liège Belgium

**Keywords:** microbiological quality, smoked fish, smoked–dried fish

## Abstract

This study aimed to assess the microbiological status of smoked fish (SF) and smoked–dried fish (SDF) processed in Benin, and to identify the contamination factors associated with these products. A total of 66 fish samples, including fresh fish and processed fish, were randomly collected from different processing sites and markets for microbial characterization using standard methods. The aerobic mesophilic bacteria (AMB) density varied from 2.9 to 9.5 Log_10_ CFU/g. *Enterobacteriaceae*, *Escherichia coli*, *Bacillus cereus*, *Clostridium perfringens*, yeasts, and molds were present in 63.9%, 27.8%, 55.6%, 58.3%, 61.1%, and 77.8% of samples, respectively, while no *Salmonella* spp., *Listeria monocytogenes,* and *Staphylococcus aureus* were found. The majority (66.7%) of SF samples and 22.2% of SDF samples were not compliant with the acceptable limit of <7.0 Log_10_ CFU/g recommended by the Health Protection Agency for AMB, whereas the *Enterobacteriaceae* counts exceeded the recommended level of 4.0 Log_10_ CFU/g for 50% of SF and 5.6% of SDF samples. Likewise, 38.9% of SF samples were not compliant for *E. coli*. Microbiological hazard analysis of practices allowed to identify the sensitive steps where hygiene measures need to be emphasized for an improved quality control.

## 1 INTRODUCTION

Fish and fishery products are an important food component for a large part of the world's population, with an average consumption level of 20.1 kg per capita (FAO, 2016). In developing countries, fish is a relatively cheap and accessible protein source, suitable for complementing high carbohydrate‐based diets of West African population (Adeyeye, Oyewole, Obadina, & Omemu, 2015; Ikutegbe & Sikoki, 2014). Among muscle food, fish is the most perishable and loses freshness after death due to autolytic and microbial spoilage (Dehghani, Hosseini, & Regenstein, 2018; Matak, Tahergorabi, & Jaczynski, 2015). In tropical regions, conservation of fresh fish remains a problem because of the lack of adequate infrastructures, and environmental and climatic conditions that contribute to its spoilage within few hours (Anihouvi, Kindossi, & Hounhouigan, 2012). To prevent fish spoilage and reduce postcapture losses, various preservation methods including frying, fermentation, drying, salting, and smoking are used (Adeyeye et al., 2015; Ikutegbe & Sikoki, 2014). Smoking consists in submitting fish to direct or indirect action of smoke during the incomplete combustion of certain trees used as fuel. Smoking of foodstuffs improves food organoleptic characteristics, induces water loss, and reduces the microbial load, thanks to heat and the presence of aromatic and bactericidal substances (Chakroborty & Chakraborty, 2017; Yusuf et al., 2015).

In Benin, fish products are the most important source of animal proteins (Kpodékon et al., 2014). Traditional smoking, one of the main methods used for fish preservation in the country (Dégnon et al., 2013), generates two types of end products, smoked fish (SF) and smoked–dried fish (SDF), used for local consumption or exported to neighboring countries.

Fish and fish products are involved in 10%–20% of foodborne diseases (Pilet & Leroi, 2011), and the presence of pathogenic bacteria such as *Staphylococcus aureus*, *Salmonella* spp., pathotypes of *Escherichia coli*, and *Listeria monocytogenes* has been reported in SF (Adeyeye et al., 2015; Ayeloja, George, Jimoh, Shittu, & Abdulsalami, 2018; Ineyougha, Orutugu, & Izah, 2015; Likongwe, Kasapila, Katundu, & Mpeketula, 2018; Nunoo & Kombat, 2013; Udochukwu, Inetianbor, Akaba, & Omorotionmwan, 2016). Another concern is the contamination by fungi. In this respect, various studies have reported the occurrence of aflatoxigenic fungi in SDF (Ayeloja et al., 2018; Babalola, Odebode, Ojomo, Ogungbemile, & Jonathan, 2018; Job, Agina, & Dapiya, 2016; Wogu & Iyayi, 2011), which under certain conditions can produce mycotoxins (Wogu & Iyayi, 2011). It is therefore necessary to take action by improving the microbiological quality of SF and SDF. The present study aims to provide insights into the microbiological status of SF and SDF processed in Benin, and to identify associated contamination factors in order to formulate suitable corrective measures.

## 2 MATERIAL AND METHODS

### 2.1 Sample collection

A total of 66 fish samples including fresh fish and processed fish were collected. Samples of SF and SDF from six fish species (*Scomber scombrus*, *Merluccius polli*, *Oreochromis niloticus*, *Cypselurus cyanopterus*, *Sphyranea baraccuda,* and *Ethmalosa fimbriata*) were randomly purchased at different processing sites and markets at Aguégués, Cotonou, Abomey‐Calavi, Comé, and Aplahoué municipalities (Benin). The samples were collected using individual sterile plastic bags and were transported under refrigeration to the laboratory.

### 2.2 Follow‐up of the manufacturing processing

The follow‐up of the processing was performed on two fish species: *S. scombrus* (smoking) and *C. cyanopterus* (smoking–drying) identified as the most used by processors, according to a previous survey (data not shown). Twelve trials were performed with three experienced processors, each performing two smoking and two smoking–drying trials. Three types of samples were collected at sensitive steps: raw fresh fish, cleaned raw fresh fish, and SF (or SDF).

### 2.3 Enumeration of the smoked and smoked–dried fish microflora

For each sample, 25 g was suspended in 225 ml of buffered peptone water (Bio‐Rad, pH 7.0 ± 0.2) and homogenized (230 rpm for 2 min) using a stomacher (Lab Blender; Seward Medical) to obtain a 1/10 dilution. Decimal dilutions were prepared in BPW as described by ISO 6887‐3 and inoculated in different media: (a) Plate Count Agar (Bio‐Rad) for aerobic mesophilic bacteria (AMB), incubated at 30°C for 72 ± 3 hr (ISO 4833‐1); (b) De Man–Rogosa–Sharpe Agar (Bio‐Rad) for lactic acid bacteria (LAB), incubated at 30°C for 72 ± 3 hr (ISO 15124); (c) Violet Red Bile Glucose (Bio‐Rad) for *Enterobacteriaceae*, incubated at 37°C for 24 ± 2 hr (ISO 21528‐2); (d) TBX Agar (Bio‐Rad) for *E. coli* incubated at 37°C for 21 ± 3 hr (ISO 16649‐2); (e) Tryptose Sulfite Cycloserine Agar (Bio‐Rad) supplemented with *Perfringens* Selective Supplement (SFP Oxoid) for *Clostridium perfringens*, incubated at 37°C for 24–48 hr (presumptive colonies confirmed with Lactose Sulfite (ISO 7937)); (f) Baird–Parker Agar (Bio‐Rad) supplemented with Rabbit Plasma Fibrinogen (Bio‐Rad) and incubated at 37°C for 24 hr (ISO 6888‐2) for *S. aureus*; and (g) Mannitol Egg Yolk Polymyxin Mossel base (Biokar Diagnostics‐Zac) with Egg Yolk (Biokar Diagnostics‐Zac) incubated at 30°C for 18–24 hr (ISO 7932) for *Bacillus cereus*. Yeasts and molds were investigated on Yeast Glucose Chloramphenicol Agar (Bio‐Rad) incubated at 25°C for 72–120 hr (ISO 7954). *L. monocytogenes* was sought on Rapid'L mono Agar (Bio‐Rad) incubated at 37°C for 24 ± 2 hr (BRD). *Salmonella* spp. were investigated on Rapid'Salmonella Agar (Bio‐Rad) after enrichment in buffered peptone water with addition of active supplement (capsules Bio‐Rad). Confirmation was performed using *Salmonella* Latex Kit (Bio‐Rad) for agglutination test according to the validated method BRD 07/11‐12/05.

### 2.4 16S rRNA sequencing

Sequencing of 16S rRNA gene was performed on fresh colonies. PCR mixture was prepared by using sterile bi‐distilled water, MgCl_2 _(25 mM), Universal primers Univ1 (10 μM) and Bact4 (10 μM), DNTP Mix (2 mM), GoTaq Flexi Buffer and GoTaq enzyme in adequate amount. Sequences of the universal primers Univ1 and Bact4 were 5′‐ACTCCTACGGGAGGCAG‐3′ and 5′‐GGCGTGTGTACAAGGCCCGG‐3′, respectively. One μl of the suspension of a fresh colony in Ringer was mixed with 49 μl PCR mixture. Amplification was performed in a C1000™ Thermal Cycler (Bio‐Rad) as follows: 5 min at 95°C, 30 cycles (1 min at 95°C, 1 min at 50°C, 1 min at 72°C) and 10 min at 72°C. Five microlitre of products was run on 0.8% agarose gel (0.5% Tris–acetate–EDTA buffer) to which 5 μl/100 ml EtBr was added. DNA bands were visualized by UV light photography.

PCR products were purified using purification kit (GenElute™ PCR Clean‐Up; Sigma‐Aldrich). Identification of the bacteria was done by comparing their 16S rRNA sequence with those in databases using www.ncbi.nlm.nih.gov/BLAST.

### 2.5 Data analysis

Statistical analyses were performed using STATISTICA 7.1. The analysis of data was performed with Student's *t* test, Mann–Whitney *U* test, one‐way analysis of variance (ANOVA), and Kruskal–Wallis ANOVA. Significant difference was established at *p* < 0.05, and means were separated using Student, Newman, and Keuls range test.

## 3 RESULTS AND DISCUSSION

### 3.1 Microbiological characteristics of SF and SDF samples

Table 1 shows the microbial loads of SF and SDF samples. AMB and LAB were the most frequent and dominant flora in both types of fish, with AMB reaching concentrations up to 9.5 and 7.8 Log_10_ (CFU/g) in the SF and SDF samples, respectively. *Enterobacteriaceae*, *E. coli*, *B. cereus, C. perfringens*, yeasts, and molds were observed in number of samples, while *Salmonella* spp., *L. monocytogenes,* and *S. aureus* were not detected.

**Table 1:** Microbial density (Log_10_ CFU/g) in SF and SDF samples (*n* = 36)

Type of fish	Parameters	Mean ± *SD*	Min.	Max.	Positive samples^a^	AL^b^	NCS
SF (*n* = 18)	AMB	7.4 ± 1.8^c^	3.8	9.5	18 (100%)	<7	12 (66.7%)
	LAB	7.0 ± 1.7^c^	3.7	9.2	18 (100%)	NE	NA
	Enterobacteriaceae	4.4 ± 2.7^c^	<1	8.2	16 (88.9%)	4	9 (50.0%)
	*Escherichia coli*	2.8 ± 2.5^c^	<1	6.8	8 (44.4%)	2	7 (38.9%)
	*Bacillus cereus*	1.5 ± 1.5^c^	<1	5.1	5 (27.8%)	5	2 (11.1%)
	*Clostridium perfringens*	1.1 ± 1.2^c^	<1	5.7	13 (72.2%)	4	1 (5.6%)
	Yeasts	3.5 ± 1.8^c^	<1	5.7	17 (94.5%)	NE	NA
	Molds	3.3 ± 1.9^c^	<1	6.0	13 (72.2%)	NE	NA
	*Staphylococcus aureus*	<1	<1	<1	0 (0%)	2	0 (0%)
	*Listeria monocytogenes*	<1	<1	<1	0 (0%)	2	0 (0%)
	*Salmonella* spp.	Abs	NA	NA	0 (0%)	Abs	0 (0%)
SDF (*n* = 18)	AMB	4.8 ± 1.7^c^	2.9	7.8	18 (100%)	<7	4 (22.2%)
	LAB	4.1 ± 1.9^c^	<1	7.3	17 (94.4%)	NE	NA
	Enterobacteriaceae	1.5 ± 1.4^c^	<1	5.1	7 (38.9%)	4	1 (5.6%)
	*E. coli*	0.8 ± 0.2^c^	<1	1.3	2 (11.1%)	2	0 (0.0%)
	*B. cereus*	2.8 ± 0.9^c^	<1	4.4	15 (83.3%)	5	0 (0.0%)
	*C. perfringens*	1.1 ± 0.5^c^	<1	2.1	8 (44.4%)	4	0 (0.0%)
	Yeasts	1.0 ± 0.6^c^	<1	2.7	5 (27.8%)	NE	NA
	Molds	2.1 ± 1.1^c^	<1	4.1	15 (83.3%)	NE	NA
	*S. aureus*	<1	<1	<1	0 (0%)	2	0 (0%)
	*L. monocytogenes*	<1	<1	<1	0 (0%)	2	0 (0%)
	*Salmonella* spp.	Abs	NA	NA	0 (0%)	Abs	0 (0%)

Abs, absence in 25 g; AMB, aerobic mesophilic bacteria; LAB, lactic acid bacteria; *n*, number of samples analyzed; NA, not applicable; NCS, noncompliant samples; NE, not established; *SD*, standard deviation; SF, smoked fish.

a Positive samples = samples in which the number of detected colonies is ≥ 1.

b AL = acceptable limit according to Health Protection Agency (2009).

c For each parameter, mean values followed by different letters indicate that they differ significantly between smoked versus SDF (*p* < 0.05).

The minimum and maximum values recorded for each criterion showed important variability within samples of each type of product. This variation can be explained by the fact that the samples were collected from various processors and sellers where the quality of the raw material varied, as well as handling and hygiene practices. Also, the density of AMB*, Enterobacteriaceae,* and *E. coli* was significantly higher (*p *< 0.05) in SF than in SDF (Table 1). This is probably due to the fact that SF samples have a higher moisture content (61 ± 11%) than the SDF ones (24 ± 11%). Also, in daily practices, SF are often more handled than SDF both by processors and customers.

Furthermore, 66.7%, 50.0%, 38.9%, 11.1%, and 5.6% of SF were not compliant with the stipulated limits for seafood products (Health Protection Agency, 2009) regarding AMB, *Enterobacteriaceae*, *E. coli*, *B. cereus,* and *C. perfringens*, respectively. Similarly, 22.2% and 5.6% of SDF samples were not compliant with the acceptable limits for AMB and *Enterobacteriaceae*, respectively. The high load of AMB is likely due to a high contamination level of the raw material, and these microorganisms were not fully eliminated during the smoking treatment. The postprocess handling and storage conditions are also potential sources of renewed pollution of the processed fish, as reported by Kpodékon et al. (2014).

Thermosensitive bacteria like *Enterobacteriaceae* are used as indicators of hygiene conditions and contamination of food after cooking (Health Protection Agency, 2009). The detection of *E. coli* in ten samples (eight SF and two SDF) also suggested a contamination by fecal matter from animal or human origin during postsmoking handling.

The presence of *B. cereus* and *C. perfringens* at levels above the permitted limits may constitute a hazard to consumer's health. *B. cereus* and *C. perfringens* are foodborne pathogens mostly evocated in gastrointestinal diseases in developed countries (Dierick et al., 2005; EFSA & ECDC, 2016; Lindström, Heikinheimo, Lahti, & Korkeala, 2011). The presence of large numbers of molds, especially in SDF, also poses a risk, since they may produce mycotoxins during long‐term storage (Job et al., 2016; Wogu & Iyayi, 2011).

As indicated in Table 2, no significant (*p *> 0.05) differences were observed between the microbial counts of samples from processing sites and those collected from markets regarding AMB, *Enterobacteriaceae*, *B. cereus*, *C. perfringens,* and yeasts in the case of SF. However, *E. coli* counts in SF samples collected from processing sites were significantly (*p *< 0.05) higher than those from markets. This could be explained by the additional smoking of leftover products intended to extend their shelf life. Likewise, mold counts in SF samples collected from markets were significantly higher (*p *< 0.05) than those from processing sites.

**Table 2:** Microbial density (Log_10_ CFU/g) of SF and smoked–dried fish (SDF) according to sampling places

Type of fish	Parameters	Processing site (*n* = 9)	Market (*n* = 9)
SF (*n* = 18)	AMB	7.7 ± 1.6^a^	7.0 ± 1.9†, a
	Enterobacteriaceae	4.9 ± 2.9^a^	4.0 ± 2.7^a^
	*Escherichia coli*	4.1 ± 2.6^a^	1.8 ± 2.0^b^
	*Bacillus cereus*	1.7 ± 1.6^a^	1.6 ± 1.8^a^
	*Clostridium perfringens*	1.3 ± 1.5^a^	0.8 ± 0.2^a^
	Yeasts	1.4 ± 0.7^a^	3.7 ± 1.7^a^
	Molds	2.6 ± 0.9^a^	4.2 ± 1.5^b^
Smoked–dried fish (*n* = 18)	AMB	3.9 ± 1.4^a^	5.7 ± 1.5^b^
	Enterobacteriaceae	1.4 ± 1.2^a^	1.7 ± 1.6^a^
	*E. coli*	<1	<1
	*B. cereus*	1.6 ± 0.8^a^	2.3 ± 0.9^a^
	*C. perfringens*	1.0 ± 0.4^a^	1.2 ± 0.5^a^
	Yeasts	<1	1.4 ± 0.8
	Molds	1.6 ± 1.2^a^	2.6 ± 0.9^a^

Note

AMB, aerobic mesophilic bacteria; *n*, number of samples analyzed; SF, smoked fish.

^†^ Mean ± standard deviation.

^a,b^ for each parameter, mean values followed by different letters indicate that they differ significantly between processing site versus market (*p* < 0.05).

### 3.2 Identification of MRS‐associated bacteria

Bacteria grown on MRS constituted the dominant flora of both SF and SDF samples. Although MRS is a nonselective medium for LAB, it can also promote the growth of other microorganisms. 16S rRNA sequencing was performed on 12 CFU isolated on MRS. Five LAB species (*Lactococcus garvieae*, *Pediococcus acidilactici*, *Weissella paramesenteroides*, *Enterococcus faecalis*, and *Enterococcus hirae*) together with *Klebsiella pneumoniae* and *Staphylococcus piscifermentans* were found.

*Klebsiella pneumoniae* is known to possess histidine decarboxylase activity, enabling the bacterium to produce histamine in fish products (Visciano, Schirone, Tofalo, & Suzzi, 2012), which causes various health disorders to humans (Maintz & Novak, 2007). *L. garvieae* is found in aquatic environments (marine and freshwater aquaculture) and is a pathogen for fish (Vendrell et al., 2006). Wang et al. (2007) reported that it can be pathogenic for human with gastrointestinal disorder. *E. faecalis* has also been reported to cause endocarditis and diverse infections. Its transmission is nosocomial, but can be also done by food (Oprea & Zervos, 2007). Thus, beside the conventional microorganisms investigated for assessing the safety of ready‐to‐eat foods, SF and SDF samples also contained other potential pathogenic microorganisms exposing consumers to foodborne diseases. However, some of these bacteria can have positive effects. For instance, *P. acidilactici* produces a bacteriocin (Bacteriocin PA‐1 or Pediocin AcH), which has an inhibitory effect on *L. monocytogenes* (Nieto‐Lozano et al., 2010), and *W. paramesenteroides* secretes a bacteriocin with a broad spectrum of inhibition of spoilage bacteria and food pathogens such as *Salmonella* typhimurium, *Vibrio parahaemolyticus*, or *L. monocytogenes* (Pal & Ramana, 2010).

### 3.3 Changes in microbial loads during the processing of SF and SDF

Table 3 shows the evolution of the microbial loads during the processing of SF. AMB load in the raw frozen fish decreased significantly (*p *< 0.05) after the washing step (from 7.1 ± 0.5 to 6.2 ± 0.2), but remained stable after the smoking step (6.5 ± 0.4), close to the acceptable limit (<7 Log_10_ CFU/g). This is surprising since a significant reduction in the microbial load of the fish was expected after the heat treatment. In addition, there were no significant (*p *> 0.05) changes in microbial loads for *Enterobacteriaceae*, *B. cereus*, yeasts, and molds during processing. Furthermore, potentially pathogenic organisms such as *E. coli*, *C. perfringens*, *S. aureus,* and *Salmonella* spp. were not detected in both fresh and SF samples.

**Table 3:** Microbial load (Log_10_ CFU/g) during the processing of SF *Scomber scombrus*

Parameters	Fresh fish (*n* = 3)	Cleaned fresh fish (*n* = 3)	SF (*n* = 6)
AMB	7.1 ± 0.5^a^	6.2 ± 0.2^b^	6.5 ± 0.4^†b^
Lactic acid bacteria	4.6 ± 0.7^a^	4.5 ± 0.4^a^	5.5 ± 0.5^b^
Enterobacteriaceae	1.7 ± 1.3^a^	<1	1.8 ± 1.8^a^
*Escherichia coli*	<1	<1	<1
*Bacillus cereus*	<1	1.3 ± 0.5^a^	0.9 ± 0.4^a^
*Clostridium perfringens*	<1	<1	<1
Yeasts	1.0 ± 0.6^a^	<1	1.9 ± 0.8^a^
Molds	2.2 ± 1.2^a,b^	1.5 ± 0.7^a^	2.7 ± 0.5^b^
*Staphylococcus aureus*	<1	<1	<1
*Listeria monocytogenes*	<1	<1	<1
*Salmonella* spp.	Abs	Abs	Abs

Note

Legend as for Table 2.

Hot smoking as carried out in traditional processing units can induce a reduction in the microbial contamination comparable to pasteurization (Plahar, Nerquaye‐Tetteh, & Annan, 1999). During the follow‐up trials, temperature values recorded in the core of fish remained above 70°C during the last 30 min of the average duration of 90 min of smoking (data not shown). Since this temperature is expected to reduce the microbial load, the hypothesis of recontamination of the product during and after smoking is therefore likely. Indeed, the follow‐up trials revealed many practices that could contribute to fish recontamination: (a) processors do not wear appropriate clothes during processing (no clean apron and charlotte) (b) the processing is performed in unhygienic environment where the product is exposed to dust and flies, (c) processors use wastewater from raw fish washing to cool their hands, and (d) cement paper or frozen fish wrap is used to cover the end products.

Table 4 shows the evolution of the microbial loads during the processing of SDF. AMB and LAB counts decreased significantly (*p *< 0.05) at the end of the smoking–drying period (from 7.4 ± 0.8 to 5.1 ± 1.1 and 6.1 ± 2.0 to 3.9 ± 1.1 Log_10_ CFU/g, respectively). Moreover, *Enterobacteriaceae* were not detected at the end of the smoking–drying period. However, *B. cereus*, yeasts, and molds counts were not reduced significantly (*p *> 0.05), which could be explained by a recontamination of the product by these microorganisms during postprocess handling. As for SF, potential pathogenic bacteria such as *L. monocytogenes*, *Salmonella* spp., *E. coli,* and *C. perfringens* were not detected along the process except *S. aureus*, which was detected in low amount in fresh fish (1.0 Log_10_ CFU/g) and eliminated after the cleaning step.

**Table 4:** Microbial load (Log_10_ CFU/g) during the processing of smoked–dried fish (SDF) *Cypselurus cyanopterus*

Parameters	Fresh fish (*n* = 3)	Cleaned fresh fish (*n* = 3)	SF (*n* = 6)	SDF (*n* = 6)
AMB	7.4 ± 0.8^†a^	6.5 ± 0.3^a,b^	6.0 ± 1.2^a^	5.1 ± 1.1^b^
Lactic acid bacteria	6.1 ± 2.0^a^	4.4 ± 2.2^a,b^	5.2 ± 1.1^a,b^	3.9 ± 1.1^b^
Enterobacteriaceae	1.7 ± 1.0^a^	2.1 ± 1.2^a^	1.8 ± 1.8^a^	<1
*Escherichia coli*	<1	<1	<1	<1
*Bacillus cereus*	1.2 ± 0.9^a^	<1	1.0 ± 0.4^a^	1.4 ± 0.8^a^
*Clostridium perfringens*	<1	<1	<1	<1
Yeasts	1.8 ± 1.0^a^	1.3 ± 0.7^a^	1.9 ± 1.6^a^	0.9 ± 0.5^a^
Molds	2.3 ± 0.8^a^	1.7 ± 1.3^a^	2.5 ± 1.7^a^	1.3 ± 0.9^a^
*Staphylococcus aureus*	1.0 ± 0.3	<1	<1	<1
*Listeria monocytogenes*	<1	<1	<1	<1
*Salmonella* spp.	Abs	Abs	Abs	Abs

Note

Legend as for Table 2.

## 4 CONCLUSION

This study revealed that SF and SDF processed in Benin are not always of satisfactory microbiological status and represent potential sources of foodborne diseases. The quality of raw material, poor hygiene practices, and inappropriate handling practices during processing and selling is factors that contribute to the unsatisfactory microbiological quality of these products. Processors and sellers should be trained on good hygiene and handling practices in order to produce a safe and sound product for consumption.

## CONFLICT OF INTEREST

The authors have no conflicts of interest to declare.

## ETHICAL REVIEW

This study does not involve any human or animal testing.

## INFORMED CONSENT

Written informed consent was obtained from all study participants.

## References

[fsn31030-bib-0001] Adeyeye, S. A. O. , Oyewole, O. B. , Obadina, A. O. , & Omemu, A. M. (2015). Microbiological assessment of smoked silver catfish (*Chrysichthys nigrodigitatus*). African Journal of Microbiology Research, 5, 1821–9.

[fsn31030-bib-0002] Anihouvi, V. B. , Kindossi, J. M. , & Hounhouigan, J. D. (2012). Processing and quality characteristics of some major fermented fish products from Africa: A critical review. International Research Journal of Biological Sciences, 1, 72–84.

[fsn31030-bib-0003] Ayeloja, A. A. , George, F. O. A. , Jimoh, W. A. , Shittu, M. O. , & Abdulsalami, S. A. (2018). Microbial load on smoked fish commonly traded in Ibadan, Oyo State, Nigeria. Journal of Applied Science and Environmental Management, 22, 493–497. 10.4314/jasem.v22i4.9

[fsn31030-bib-0004] Babalola, B. J. , Odebode, J. A. , Ojomo, K. Y. , Ogungbemile, O. A. , & Jonathan, S. G. (2018). Mycological evaluation and nutritional composition of smoked‐dried fish from Igbokoda market in Ondo State, Nigeria. Archives of Basic and Applied Medicine, 6, 51–53.

[fsn31030-bib-0005] Chakroborty, T. , & Chakraborty, C. S. (2017). Comparative analysis of nutritional composition and microbial quality of salt‐smoke‐dried mirror carp (*Cyprinus carpio* var. *specularis*) during storage at 22–28°C and 4°C. International Journal of Food Science and Nutrition, 1, 86–89.

[fsn31030-bib-0006] Dégnon, R. G. , Agossou, V. , Adjou, E. S. , Dahouénon‐Ahoussi, E. , Soumanou, M. M. , & Sohounhloué, D. C. K. (2013). Evaluation de la qualité microbiologique du chinchard (*Trachurus trachurus*) au cours du processus de fumage traditionnel. Journal of Applied Bioscience, 67, 5210–5218.

[fsn31030-bib-0007] Dehghani, S. , Hosseini, S. V. , & Regenstein, J. L. (2018). Edible films and coatings in seafood preservation: A review. Food Chemistry, 240, 505–513. 10.1016/j.foodchem.2017.07.034 28946304

[fsn31030-bib-0008] Dierick, K. , Van Coillie, E. , Swiecicka, I. , Meyfroidt, G. , Devlieger, H. , Meulemans, A. , … Mahillon, J. (2005). Fatal family outbreak of *Bacillus cereus*‐associated food poisoning. Journal of Clinical Microbiology, 43, 4277–4289. 10.1128/JCM.43.8.4277-4279.2005 16082000 PMC1233987

[fsn31030-bib-0009] EFSA & ECDC (2016). The European Union Summary Report on trends and sources of zoonoses, zoonotic agents and food‐borne outbreaks in 2015. EFSA Journal, 14, 4634. 10.2903/j.efsa.2017.5077 PMC7009962 32625371

[fsn31030-bib-0010] FAO (2016). Food and Agriculture Organization of the United States. The State of World's Fisheries and Aquaculture. Retrieved from http://www.fao.org/publications/sofia/2016/en/

[fsn31030-bib-0011] Health Protection Agency (2009). Guidelines for accessing the microbiological safety of ready‐to‐eat foods. London, UK: Health Protection Agency.

[fsn31030-bib-0012] Ikutegbe, V. , & Sikoki, F. (2014). Microbiological and biochemical spoilage of smoke‐dried fishes sold in West African open markets. Food Chemistry, 161, 332–336. 10.1016/j.foodchem.2014.04.032 24837959

[fsn31030-bib-0013] Ineyougha, E. R. , Orutugu, L. A. , & Izah, S. C. (2015). Assessment of microbial quality of smoked *Trachurus trachurus* sold in some markets of three South‐south States, Nigeria. International Journal of Food Research, 2, 16–23.

[fsn31030-bib-0014] Job, O. M. , Agina, E. S. , & Dapiya, S. H. (2016). Occurrence of aflatoxigenic fungi in smoke‐dried fish sold in Jos metropolis. British Microbiology Research Journal, 11, 1821–1827. 10.9734/BMRJ/2016/21465

[fsn31030-bib-0015] Kpodékon, M. , Sessou, P. , Hounkpè, E. , Yèhouénou, B. , Sohounhloué, D. , & Farougou, S. (2014). Microbiological quality of smoked mackerel (*Trachurus trachurus*), sold in Abomey‐Calavi township markets, Benin. Journal of Microbiology Research, 4, 175–179.

[fsn31030-bib-0016] Likongwe, M. C. , Kasapila, W. , Katundu, M. , & Mpeketula, P. (2018). Microbiological quality of traditional and improved kiln smoked catfish (*Clarias gariepinus*; Pisces; Clariidae) in Lake Chilwa Basin. Food Science & Nutrition, 7, 281–286. 30680182 10.1002/fsn3.885 PMC6341131

[fsn31030-bib-0017] Lindström, M. , Heikinheimo, A. , Lahti, P. , & Korkeala, H. (2011). Novel insights into the epidemiology of *Clostridium perfringens* type A food poisoning: Review. Food Microbiology, 28, 192–198. 10.1016/j.fm.2010.03.020 21315973

[fsn31030-bib-0018] Maintz, L. , & Novak, N. (2007). Histamine and histamine intolerance. American Journal of Clinical Nutrition, 85, 1185–1196. 10.1093/ajcn/85.5.1185 17490952

[fsn31030-bib-0019] Matak, K. E. , Tahergorabi, R. , & Jaczynski, J. (2015). A review: Protein isolates recovered by isoelectric solubilization/precipitation processing from muscle food by‐products as a component of nutraceutical foods. Food Research International, 77, 697–703. 10.1016/j.foodres.2015.05.048

[fsn31030-bib-0020] Nieto‐Lozano, J. C. , Reguera‐Useros, J. I. , Peláez‐Martínez, M. D. C. , Sacristán‐Pérez‐Minayo, G. , Gutiérrez‐Fernández, Á. J. , & de la Torre, A. H. (2010). The effect of the pediocin PA‐1 produced by *Pediococcus acidilactici* against *Listeria monocytogenes* and *Clostridium perfringens* in Spanish dry‐fermented sausages and frankfurters. Food Control, 21, 679–685. 10.1016/j.foodcont.2009.10.007

[fsn31030-bib-0021] Nunoo, F. K. E. , & Kombat, E. O. (2013). Analysis of the microbiological quality of processed *Engraulis encrasicolus* and *Sardinella aurita* obtained from processing houses and retail markets in Accra and Tema, Ghana. World Journal of Fish and Marine Sciences, 5, 686–692.

[fsn31030-bib-0022] Oprea, S. F. , & Zervos, M. J. (2007). *Enterococcus* and its association with foodborne illness In Simjee S. (Ed.), Foodborne disease (pp. 157–174). Totowa, NJ: Humana Press.

[fsn31030-bib-0023] Pal, A. , & Ramana, K. V. (2010). Purification and characterization of bacteriocin from *Weissella paramesenteroides* DFR‐8, an isolate from cucumber (*Cucumis sativus*). Journal of Food Biochemistry, 34, 932–948. 10.1111/j.1745-4514.2010.00340.x

[fsn31030-bib-0024] Pilet, M. F. , & Leroi, F. (2011). Applications of protective cultures, bacteriocins and bacteriophages in fresh seafood and seafood products In Lacroix C. (Ed.), Protective cultures, antimicrobial metabolites and bacteriophages for food and beverage bio preservation (pp. 1821–21). Cambridge, UK: Woodhead Publishing Series in Food Science, Technology and Nutrition, 201.

[fsn31030-bib-0025] Plahar, W. A. , Nerquaye‐Tetteh, G. A. , & Annan, N. T. (1999). Development of an integrated quality assurance system for the traditional *Sardinella* sp. and anchovy smoking industry in Ghana. Food Control, 10, 15–25.

[fsn31030-bib-0026] Udochukwu, U. , Inetianbor, J. , Akaba, S. O. , & Omorotionmwan, O. F. (2016). Comparative assessment of the microbiological quality of smoked and fresh fish sold in Benin City and its public health impact on consumers. American Journal of Microbiological Research, 4, 37–40.

[fsn31030-bib-0027] Vendrell, D. , Balcázar, L. J. , Ruiz‐Zarzuela, I. , de Blas, I. , Gironés, O. , & Múzquiz, L. J. (2006). *Lactococcus garvieae* in fish: A review. Comparative Immunology, Microbiology & Infectious Diseases, 29, 177–198. 10.1016/j.cimid.2006.06.003 16935332

[fsn31030-bib-0028] Visciano, P. , Schirone, M. , Tofalo, R. , & Suzzi, G. (2012). Biogenic amines in raw and processed seafood. Frontiers in Microbiology, 3, 1821–10. 10.3389/fmicb.2012.00188 PMC3366335 22675321

[fsn31030-bib-0029] Wang, C.‐Y.‐ C. , Shie, H.‐S. , Chen, S.‐C. , Huang, J.‐P. , Hsieh, I.‐C. , Wen, M.‐S. , … Wu, D. (2007). *Lactococcus garvieae* infections in humans: Possible association with aquaculture outbreaks. International Journal of Clinical Practice, 61, 68–73. 10.1111/j.1742-1241.2006.00855.x 16704679

[fsn31030-bib-0030] Wogu, M. D. , & Iyayi, A. D. (2011). Mycoflora of some smoked fish varieties in Benin City Nigeria. Ethiopian Journal of Environmental Studies and Management, 4, 36–38. 10.4314/ejesm.v4i1.4

[fsn31030-bib-0031] Yusuf, K. A. , Ezechukwu, L. N. , Faykoya, K. A. , Akintola, S. L. , Agboola, J. I. , & Omoleye, T. O. (2015). Influence of fish smoking methods on polycyclic aromatic hydrocarbons content and possible risks to human health. African Journal of Food Science, 9, 126–135. 10.5897/AJFS2014.1227

